# Ramadan fasting outcome among high-risk patients

**DOI:** 10.1186/s12882-022-02915-3

**Published:** 2022-09-05

**Authors:** Latifa Baynouna AlKetbi, Nico Nagelkerke, Amal AlZarouni, Mouza Al Kuwaiti, Mona Al Ghafli, Salama Al Qahtani, Bushra Al Kaabi, Mariam Al Kaabi, Ali Al Ahbabi, Yousif Al Zeyodi, Kholoud Al Ketheri, Khawla Al Nabooda, Khadija Al Tenaji, Ali AlAlawi, Hanan Abdelbaqi

**Affiliations:** 1 Abu Dhabi Healthcare Services, 81815 Alain, United Arab Emirates; 2 Community Medicine Department, UAEU, Alain, United Arab Emirates

**Keywords:** Chronic kidney diseases, High-risk patients, Ramadan, Fasting, Diabetes mellites

## Abstract

**Background:**

There is a growing literature on guidelines regarding Ramadan fasting for chronic kidney disease (CKD) patients. However, most studies only consider the impact of fasting on renal function. This study additionally aims to assess factors influencing Ramadan fasting in patients with CKD.

**Method:**

This is a prospective before and after cohort study. CKD patients were counseled regarding fasting and followed-up post-Ramadan for renal function status, actual fasting behavior, and other relevant outcomes.

**Results:**

Of the 360 patients who attended the pre-Ramadan consultation, 306 were reachable after Ramadan of whom 55.3% were female. Of these 306 67.1% reported that they had fasted, 4.9% had attempted to fast but stopped, and 28% did not fast at all. Of these 74 has a post-fasting kidney test. Of the patients, 68.1% had stage 3A CKD, 21.7% had stage 3B, 7.9% stage 4, and only 2% stage 5. Of those who fasted, 11.1% had a drop in Glomerular Filtration Rate (eGFR) of 20% or more. Those who did not fast (16.7%) presented a similar drop. Conversely, among the few who attempted to fast and had to stop, half showed a drop in eGFR of more than 20%. In linear regression, fasting was not associated with post-Ramadan eGFR, when controlling for age and baseline eGRF. There were 17 (5.6%) significant events, including one death. More significant events occurred among the group who fasted some of Ramadan days, 26.7% of the subjects experienced an adverse event—while 4.7% of the group who did not fast had a significant adverse event compared to 4.4% among those who fasted all Ramadan.

**Conclusion:**

Fasting was not a significant determining factor in renal function deterioration in the study’s population, nor did it have any significant association with adverse events.

## Background

All Muslims must fast in the ninth month of the Islamic Hijri year unless fasting poses health risks. Deciding whether an individual is entitled to such an exemption is the responsibility of physicians who can balance the benefit and harm to each patient. In addition to spiritual benefits, Ramadan fasting has proven metabolic benefits for healthy individuals and some high-risk groups, such as diabetics and chronic kidney disease (CKD) patients [1, 2]. Nevertheless, the effect of abstinence from water for long hours, change in meal timings, medication adjustments, poor adherence to medication due to change in eating and sleep patterns, acute metabolic disturbances, and medicine-related side-effects all are potential sources of harm for patients with chronic diseases.

Fasting had been shown not to affect the renal function of CKD patients in stable mild/moderate disease stages (stages 1–3), provided they are appropriately monitored and counseled. However, patients on hemodialysis or peritoneal dialysis have been advised not to fast; if they still choose to do so, to undergo careful weekly monitoring [3]. A recent review by Malik et al. recommended stratifying patients with CKD by disease severity and their overall condition, and advising them about fasting accordingly [3]. Similar recommendations have been published for diabetic patients as well [1]. However, these recommendations require validation. The risks or benefits of fasting, especially among higher risk and more vulnerable patients, also need to be better quantified. Studies in the literature mainly focus on specific interventions in such patients, with their renal function as the outcome. In practice, CKD contributes to the complexity of various conditions affecting individual patients and cannot be targeted in isolation.

Abu Dhabi Healthcare Services (SEHA) has a dedicated institution for kidney diseases that identifies all patients with renal impairment and assigns their care to the appropriate care provider, such as the Ambulatory Healthcare Services (AHS) centers or to the pre-dialysis or dialysis clinics [4]. The AHS is the largest network of primary healthcare services in the region and provides structured chronic diseases and population health programs [5, 6]. CKD patients are assigned to their primary care physicians (PCP) for follow-up and management. Additionally, AHS has initiated a project to counsel newly identified CKD patients on fasting before Ramadan. The project includes a consultation with the PCP about the patient’s care and management as well as one with a nephrologist when needed. If the patient decides to fast regardless of the risk, then the PCP must ensure that the patient is adequately informed about the risks and benefits of fasting and when to seek help. This study describes the follow-up of a cohort of CKD patients who were counseled about Ramadan fasting. It tracked the possible consequences of their decision to fast or not.

## Methods

This is an observational cohort study of CKD patients in Al Ain City, Abu Dhabi Emirate in the United Arab Emirates, by the Ambulatory Healthcare Services, AHS, the main primary healthcare provider in the Emirate of Abu Dhabi. A list of patients with abnormal eGFR was sent by the central CKD care department to the AHS center where they receive routine health care. From the AHS CKD list shortly before Ramadan 1441 AH/2020 CE a cohort of patients was recruited to be part of two quality improvement projects. The first project provides counseling about Ramadan fasting to high-risk patients. This project encompasses all chronic disease patients and has been conducted annually before Ramadan since 2016. The second project, also active for several years, specifically targets CKD patients and manages them based on centrally generated electronic medical records (EMR), that identify any patient with abnormal glomerular filtration rate (eGFR) tests and recall them for consultation in their AHS center.

In March 2020, before Ramadan (April–May), CKD patients were contacted and counseled on CKD management and fasting during Ramadan. Due to the movement restrictions imposed by the Coronavirus disease 2019 (COVID-19) pandemic, teleconsultation was used.

Based on these ongoing projects this study was designed to investigate the effect of fasting on patients' clinical outcomes. One month after Ramadan, the patients included in these projects received another call inquiring about any adverse events and whether they had fasted. A follow-up renal function test (eGFR and electrolytes) was ordered for patients who were successfully contacted. The subjects were patients with CKD who appeared on the March 2020 list in the SEHA CKD database, 18 years and older, of all nationalities, and with no comorbidities. The lists were generated from EMR reports that identified any patient visiting SEHA who had done a blood test and whose eGFR had been assessed. Patients with eGFR less than 60 mL/min/1.73 m^2^ were considered to have a significant renal impairment that needed the PCP to follow up; therefore, those patients' names were sent to their respective AHS centers. The patient lists were then filtered according to their catchment areas by the AHS’ central care coordination office and sent to the care coordinators in Al Ain AHS centers, who contacted the patients and booked them a teleconsultation appointment.

The consulting physicians were family medicine residents supervised by consultant family physicians. After the appointments were booked, the care coordinators sent the lists to the residents and supervisors, who discussed each patient's care plan. The next day, the residents conducted the teleconsultation while referring to the supervisor for any additional questions or remarks. The residents were then instructed to book another consultation mid-Ramadan and one after. Training on CKD guidelines during Ramadan was provided before the project started.

CKD was defined as the patient having eGFR less than 60 mL/min/1.73 m^2^. Significant rapid GFR decline was defined as eGFR loss of > 20% within 3 months after Ramadan. Although rapid GFR decline is defined as an annual eGFR loss of > 3 mL/min/1.73 m^2^ due to the short follow-up periods (within three months immediately after Ramadan), 3 mL/min/1.73 m^2^ may be a very low level for patients with higher eGFR and very detrimental for those with near-dialysis levels. This may represent different risks, so a 20% fall in eGFR was preferred to highlight the impact on renal function at all renal function levels.

The primary outcomes were the change in eGFR and the occurrence of adverse events during Ramadan. 360 patients had their first teleconsultation. In addition to standard demographic data, information on eGFR, comorbidities, renal function, and lipid profile was recorded. Only 306 answered the phone call after Ramadan, among whom 74 did the follow-up renal function test. Data on fasting status, the occurrence of significant health events, and time of admission into care were collected after Ramadan. Only events occurring during Ramadan were considered in this study and for those who did fast after their fast. The start of surveillance was from the first day in Ramadan to the end of the holy month. Those with adverse events served as cases for those without in this prospective observational study to investigate the effect of the exposure which is fasting. For the adverse events determinants analysis all 360 patients were included and for the change in eGFR only the 74 who did repeat the eGFR test were included in the later analysis.

After Ramadan, data was collected by calling patients and a chart review was conducted to complete it. Patients were called three times and were considered lost to follow-up if unsuccessful.

### Statistical methods

Originally the study was designed as a paired before-after comparison. The calculated required sample size for this purpose was 52 for a power of 80%, two-tailed alpha of 0.05, to detect changes in eGFR with an effect size of 0.4. (Paired T-test). The actual power for measuring the effect of fasting on eGRF changes is best read from the results of the regression analysis.

In addition to standard descriptive analyses and graphs, multiple linear regression was used. Post-Ramadan eGFR was the dependent variable was regressed on its baseline value in addition to other independent variables that included disease stage (> = 3B vs < 3B)), sex, age, and whether patients fasted (fasting = 1), did not fast at all (fasting = 0), or only partially (fasting = 0.5). A *P*-value of < 0.05 was considered statistically significant. The regression was repeated with non-significant covariables removed with the possible exception of fasting as its effect was a major objective of the study.

The study was approved by the SEHA Institutional Review Board.

## Results

Of the 360 patients enrolled in this cohort, 48.9% were female and 51.1% male. Most were UAE nationals (69.9%); 66.1% were in CKD stage 3A, 23.6% in stage 3B, 7.2% in stage 4, and only 2.5% were stage 5 (Table 1). Diabetes and hypertension were diagnosed in 22.8% and 9.6%, respectively. A small percentage (6.6%) had both diagnoses.

**Table 1:** Subject characteristics

A. All Subjects in the study					
		Age in years			
		<=40	41–59	>=60	Total
Gender	Female	3(21.4)	23(41.1)	139(52.7)	176(48.9)
	Male	11(78.6)	33(58.9)	125(47.3)	184(51.1)
Nationality	UAE	7(50)	32(57.1)	191(72.3)	249(69.1)
	Non-UAE	7(50)	24(42.9)	73(27.7)	111(30.8)
CKD Stage	2	0	0	2(0.8)	2(0.6)
	3A	7(50)	40(71.4)	174(65.9)	238(66.1)
	3B	3(21.4)	9(16.1)	68(25.8)	85(23.6)
	4	2(14.3)	6(10.7)	15(5.7)	26(7.2)
	5	2(14.3)	1(1.8)	5(1.5)	9(2.5)
Hypertension		1(7.1)	5(8.9)	26(9.8)	32(9.6)
Diabetes Mellitus		2(14.3)	7(12.5)	67(25.4)	76(22.8)
Diabetes and Hypertension		0	3(5.4)	19(7.2)	22(6.6)
Total		14	56	264	360^a^
B. Subjects with completed eGFR lab results (before and after Ramadan)					
		Age in years			
		<=40	41–59	>=60	Total
Gender	Female	0 (0)	4 (40)	24 (42.1)	28 (40.6)
	Male	2(100)	6 (60)	33 (57.9)	41(59.4)
Nationality	UAE	1 (50)	9 (90)	47 (82.5)	57(82.6)
	Non-UAE	1 (50)	1 (10)	10 (17.5)	12 (17.4)
CKD Stage	3	2 (100)	10 (100)	53 (93)	65 (94.2)
	4, 5	0 (0)	0 (0)	4 (7)	4 (5.8)
Hypertension		0 (0)	1 (10)	3 (5.3)	4 ( 5.8)
Diabetes Mellitus		0 (0)	0 (0)	11 (19.3)	11 (15.9)
Diabetes and Hypertension		0 (0)	1 (10)	12 (21.1)	13 (18.8)
Total		2	10	57	69

Age missing in 5 subjects

^a^Age of 11 females missing

**Fig. 1:**
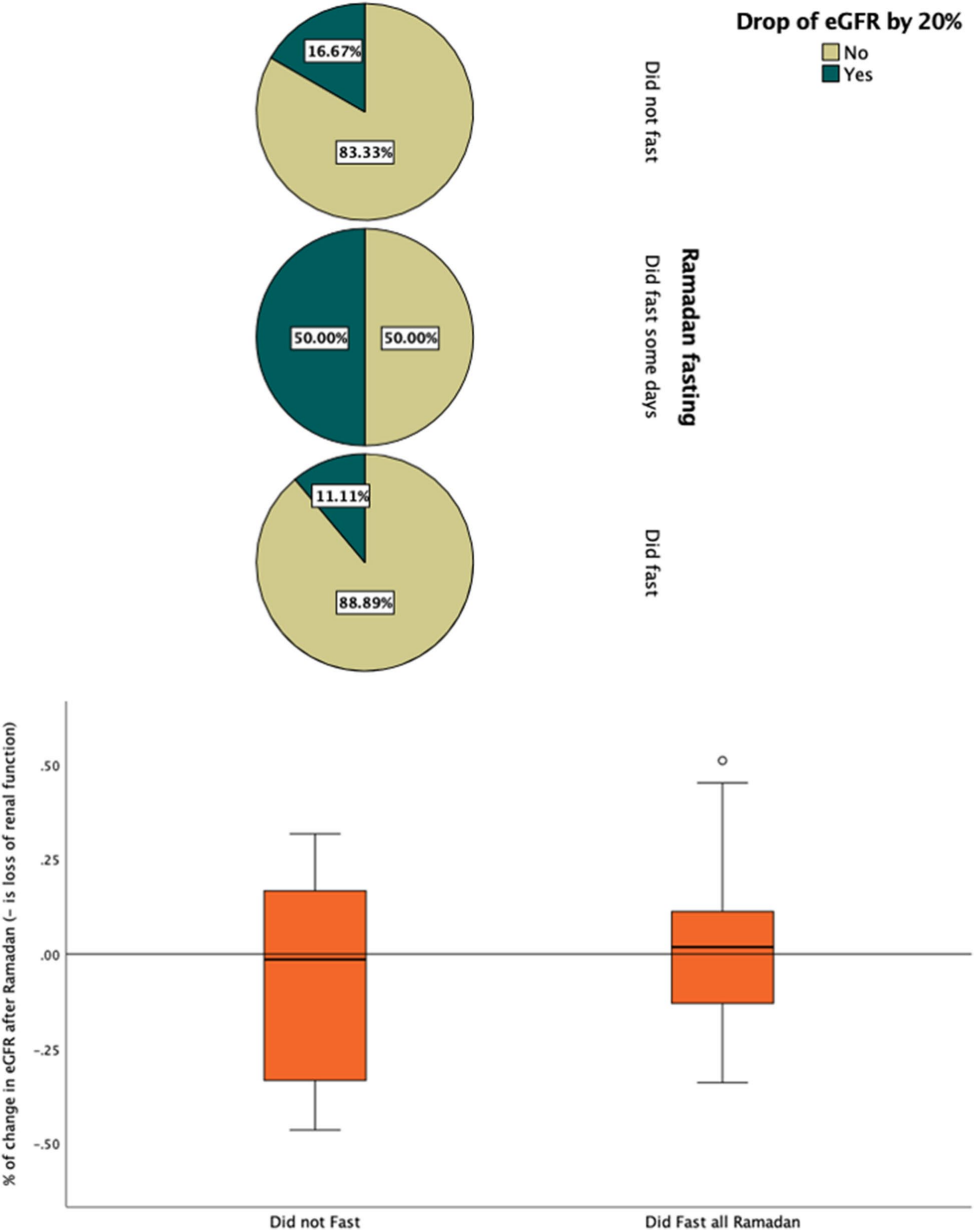
Change in eGFR after Ramadan in the three groups: those who fasted throughout Ramadan, those who attempted to fast but had to stop, and those who did not fast. **A** Drop in eGFR of 20% among the three groups. **B** Box plot of the change in percentage of eGFR in two groups: those who fasted and those who did not

Of the 360 patients who attended the pre-Ramadan consultation, 306 were reachable after Ramadan. Of these, 67.1% reported that they had fasted, 4.9% had attempted to fast but had to stop, and 28% did not fast a single day. The latter were mainly in the more severe category of CKD, stages 4 and 5. Of these 306 patients reached through phone calls after Ramadan, only 74 underwent a renal function test within SEHA. Figure 1 shows the prevalence of a >  = 20% drop in eGFR after Ramadan among the three groups. It shows that among patients fasting during Ramadan, 11.1% experienced a drop in eGFR of 20% or more, while among those who did not fast, 16.7% experienced a similar drop. Among the few who attempted to fast and had to stop, half experienced a drop in eGFR of more than 20%. The boxplot in Fig. 1B shows these changes among the two groups those who did fast and those who either fasted somedays or all Ramadan and it demonstrates the lack of significant difference between the two groups.

**Fig. 2:**
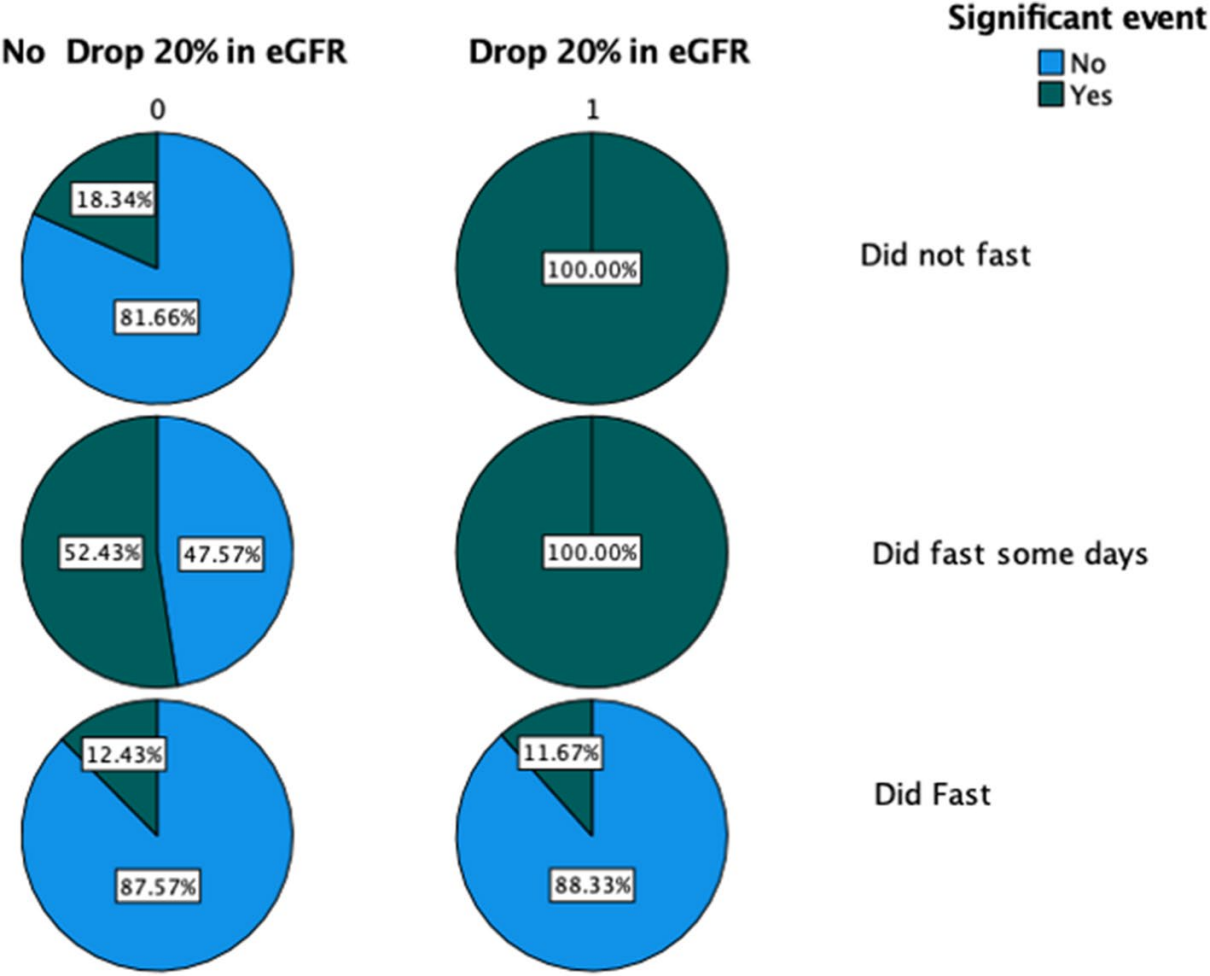
Occurrence of significant adverse event and drop in eGFR in relation to fasting status

Using linear regression of post-Ramadan eGFR in relation to studied variables showed that neither sex nor disease stage was a significant predictor of post-Ramadan eGFR. With these two variables excluded we obtained a highly (positive) significant association with baseline eGFR value (B = 1.138, *p* < 0.001), a significant negative association with age (B = -0.2, *p* = 0.03), and a non-significant positive association with fasting, Table 2. Thus, there appears to be no significant adverse effect of fasting on eGFR post-Ramadan. There were 17 (5.6%) significant adverse events, including one death. The majority of these events (15) were in the age group 60 years or older. In addition, six COVID-19 infections occurred, all of whom recovered. More significant events were among the group that chose to fast; 26.7% suffered adverse events as compared to only 4.4% in the non-fasting group and 4.4% in the fasting group (Fig. 2). It is clear that patients who were unable to fast and had higher declines in renal function had higher rates of adverse events. While fasting was not a risk for adverse event (Table 3), when those who attempted to fast were combined with those who fasted, the incidence of an adverse event was 5.9% (Table 3). This is compared to 4.4% among those who never attempted to fast. The incidence of admission among those who did not fast and among those who did fast or attempted to fast, were 11.8% vs 10% respectively. Of note is that the group who attempted but could not continue to fast had the highest hospitalization rate, 20% and the highest rate of adverse events 26%. Overall, there was no difference between the two groups; group of subjects who did not fast and the group of subjects who did fast or who attempted to fast with regards to the occurrence of a significant event or admission 16.5%, 15.9% respectively, Table 3. Worth noting that although of the 38 admissions among all subjects, 31 being in the age group 60 years or older, using logistic regression, age and other studies determinants were not significantly associated with higher risk of admissions. Covariables included in this analysis were CKD stage, sex, fasting status, Diabetes, hypertension, and decline in eGFR during the previous year.

**Table 2:** Linear regression of post-Ramadan eGFR in relation to studied variables

	Unstandardized Coefficients	Std. Error	Standardized Coefficients	t	*P* value
	B		Beta		
eGFR result before Ramadan	1.138	0.112	0.755	10.142	< 0.001
AGE	-0.204	0.092	-0.159	-2.222	0.030
Did not fast	Reference				
Did fast	1.541	3.736	0.036	0.413	0.681
Did fast some days	-6.697	6.062	-0.094	-1.105	0.273

**Table 3:** Prevalence of significant outcomes in relation to the actual fasting behavior

	Did not fast N (%)	Who fasted or attempted to fast Ramadan			
		Did fast some days N (%)	Did fast All Ramadan N (%)	Total of those who did fast or attempted N (%)	Total of all subjects
Admitted to hospital (total 32)	10(11.8)	3(20)	19(9.3)	22(10)	32
Had a significant adverse health event (total 17)	4(4.7)	4(26.7)	9(4.4)	13(5.9)	17
Admitted to hospital or had a significant adverse health event	14 (16.5)	7(46.7)	28(13.7)	35(15.9)	49
Total number of subjects within actual fasting behavior category	85	15	204	219	304

## Discussion

All patients with eGFR less than 60 were advised to consider not fasting during Ramadan. Furthermore, all stages 4 and 5 patients were strongly advised against fasting. Yet, fasting remained a patient's individual choice. Knowledge about risks and warning signs to be heeded may have affected outcomes in this cohort. Nevertheless, the observed effects of fasting in this high-risk group suggest several conclusions. Most importantly, fasting appeared not to be associated with disease progression as reflected in (changes in) eGFR value.. Although the number of adverse events was not significantly affected by fasting, the relatively high rate of adverse events (17, including one death), despite not fasting seems to support this advice. In our study, diabetes was not associated with worse outcomes, unlike an earlier study that found both diabetes mellitus and proteinuria to be independent predictors of renal dysfunction [7]. Clearly, this deserves further exploration and glycemic control and duration of diabetes may be factors of interest in future studies.

Our findings overall support the recommendation in international guidelines regarding fasting during Ramadan in CKD patients [3]. Nevertheless, it highlights other areas of importance to consider when suggesting decisions with regards to Ramadan fasting. There is a need to consider the patient’s overall risk rather than to rely on single lab value or diagnosis. The interpretation of the findings regarding increased admissions and adverse events in the group that attempted to fast but had to stop, and the similar occurrence of admissions among those who fasted and those who did not cannot be interpreted without considering the major influence of confounders. There may be a group who did not fast due to some health condition or those with bad health who insisted on fasting but could not. This could be better studied if risk assessment and stratification were done before Ramadan and prospectively outcome were assessed, which is the subject of an ongoing study.

A limitation of this study is its observational design, which does not allow to infer causality from association. Both self-selection and confounding by unobserved variables may have affected the choice to fast and the outcomes. An improvement on our methodology would be to reduce confounding by measuring and adjusting for more prognostic variables. Another limitation of our study is its limited follow-up, without which it is impossible to assess long-term outcomes. Selection bias could have occurred as patients were clearly not selected randomly; rather, they were tested either routinely or due to health issues that might affect the representativeness of our patients. Nevertheless, patients were recruited from a community database and not hospitals—therefore, they were probably reasonably representative of the community, although a more complex or a higher risk group of the community.

Important lessons learned from this project to integrate in delivering the same project for the coming Ramadan include introducing more risk assessment tools and increasing the intensity of follow-ups by the PCP for very high-risk patients.

## Conclusion

Fasting was not a factor in renal function deterioration or in other adverse events in patients with CKD.

## Data Availability

Data is available on request sent to the corresponding author.

## Abbreviations

CKD: Chronic Kidney Diseases
GFR: Glomerular Filtration Rate
PCP: Primary Care Physicians
EMR: Electronic Medical Records
COVID-19: Coronavirus Disease 2019
